# Not only static: Stabilization manoeuvres in dynamic exercises – A pilot study

**DOI:** 10.1371/journal.pone.0201017

**Published:** 2018-08-08

**Authors:** Giedrė Vaičienė, Kristina Berškienė, Agne Slapsinskaite, Vilma Mauricienė, Selen Razon

**Affiliations:** 1 Institute of Sports, Lithuanian University of Health Sciences (LUHS), Kaunas, Lithuania; 2 Health Research Institute, LUHS, Kaunas, Lithuania; 3 Department of Kinesiology, West Chester University, West Chester, PA, United States of America; University of Illinois at Urbana-Champaign, UNITED STATES

## Abstract

This study examined characteristics of trunk muscles electrical activity in young adults performed in the course of static and dynamic trunk muscles strengthening exercises using different lumbar spine stabilization manoeuvres. Twenty young adults (M_age_ = 25.5 SD = 2.91) participated in this study. Of the 20, 11 subjects (5 males and 6 females) reported no history of pain, 9 subjects (5 males and 4 females) reported lower back pain (LBP) within the last three months. Subjects performed lumbar spine stabilization manoeuvres (abdominal bracing (AB) and abdominal hollowing (AH)) with static and dynamic abdominal muscles strengthening exercises (i.e., plank, side-bridges, and curl-ups). Noraxon Telemyo twelve channel electromyography device (Noraxon USA, Inc.) was used to record EMG data from rectus abdominal muscle (RA), external oblique muscles (EO), internal oblique muscles (IO), and erector spine muscles (ES). In static exercises such as side-bridge exercise, significantly higher RA muscle electrical activity was recorded with AB manoeuvre compared to AH manoeuvre both on the right side and left side respectively (Z = -2.17; p = 0.03; Z = 3.40; p = 0.001). In dynamic exercises such as curl-up exercise, during the lifting phase, median value of RA muscle activity with AB was significantly higher than with AH (Z = -2.315; p = 0.021). Median value of IO muscles activity with AH was significantly higher than with AB (Z = -3.230; p = 0.001). Our findings indicated that although surface muscles are more activated with AB manoeuvre exercises, deep abdominal muscles are more activated in exercises with AH manoeuvre. These findings can help practitioners design interventions to integrate AH manoeuvre for benefiting persons with lumbar instability.

## Introduction

Pain is generally defined as the perception of a distressing experience associated with actual or potential tissue damage that entails sensory, cognitive, emotional, and social components [1]. Specifically, lower back pain (LBP) refers to muscle tension, stiffness or discomfort localized below the rib cage and above the upper buttock folds [2]. LBP is very common and remains a frequent cause of disability in modern society [3, 4]. To date, the prevalence of LBP is around 10–30% in the general population [5], with chronic non-specific LBP making up for close to 85% of all back pain cases [6]. Despite the common occurrence of pain; however, studies are yet to examine the influence and effects of different lumbar spine stabilization manoeuvres in dynamic exercise.

There are a number of risk factors that can contribute to LBP. Among these are long-term, static work, obesity, older age, depression, spinal pathology etc. [7]. The identification of the exact cause of pain in the lumbar spine remains complicated. To date, there is consensus that the lack of mechanical stability in the lumbar spine results in inappropriate muscle activation patterns and contributes to the appearance of non-specific LBP symptoms [8]. Additionally, some studies confirm that patients with LBP often have a dysfunctional muscle function [9–12].

Clinical guidelines recommend physical exercises as one modality for the treatment of lumbar spine issues [13]. Appropriate physical exercises [14] can help restore the stability of the lumbar spine which is defined as the ability of the active and passive waist—pelvis structures to maintain the proper trunk, pelvis position, balance, and control of static and dynamic movements [15]. The ‘sufficient stability’ is a term that designates the determination of how much muscular stiffness is necessary for stability, together with a modest amount of extra stability to form a margin of safety [16]. Decreased motor control of the lumbar spine during movement can contribute to tissue sprain and cause chronic LBP [17]. In general, surface or global muscles including rectus abdominal muscle (RA), external oblique muscles (EO) and erector spine muscles (ES) are responsible for trunk and pelvic motion [18, 19]. Deep abdominal muscles, also known as local muscles, such as internal oblique muscles (IO), transversus abdominus muscles (TrA), and lumbar multifidus muscles (LM) stabilize the spine by joining the thoracolumbar fascia and increase intra-abdominal pressure [18]. Finally, it is important to understand that the spinal stabilization system consists of several interacting elements: a) non-muscular control (neural element), b) passive subsystems (bone and ligament elements) and c) active subsystem (muscle element). Although there are some studies looking into the effects of lumbar spine stabilization manoeuvres on trunk muscle activity in different starting positions [20–22] or using loads [23, 24], there is a lack of data showing trunk muscle activity performing lumbar spine stabilization manoeuvres with static and dynamic abdominal muscle strengthening exercises.

Abdominal hollowing (AH) and abdominal bracing (AB) manoeuvres are used in rehabilitation and exercise training programs [21]. The purpose of these manoeuvres is to stabilize the lumbar spine prior to accepting dynamic loads, thus protecting the spine from trauma [23]. AH manoeuvre, also known as abdominal in-drawing or abdominal drawing-in manoeuvre (ADIM) [25,26], primarily activates deep trunk muscles with minimal surface muscles activation [27]. This manoeuvre consists of a lower abdominal hollowing, where the individual draws the abdomen down toward the spine (in the direction of the vertebra) and a neutral position is maintained in the lumbar spine, thus activating TrA and IO muscles [28]. Specifically, IO and TrA muscles work together to increase intra-abdominal pressure by connecting to the thoracolumbar fascia and the increased intra-abdominal pressure gives firmness to the spine [18]. Meanwhile, AB is defined as abdominal and lower back muscles co-activation without abdominal drawing in or distension [13]. From a biomechanical point of view, it is believed that all trunk muscles play an important role in achieving spinal stability and ideally all the muscles should work harmoniously towards this goal. According to AB approach however, one or two muscles should not be specifically trained, on the contrary, stabilization exercises should achieve a global co-activation [28]. Of specific interest herein, only one muscle with inappropriate activation amplitude can lead to instability (if passive stiffness is not sufficient), or at least unstable behaviour could result from inappropriate activation at lower applied loads [16].

The surface electromyography is a widely used method for determining muscle activity during exercise [28]. It provides information about the specific activity of a particular muscle activation during exercise, as well as the optimal position required for exercise [29]. To that end, exercises with muscle electrical activity greater than 50% of maximal isometric voluntary contraction (MVC) are sufficient to increase the muscle force [29, 30]. Although the lumbar spine stabilization manoeuvres are used in health promotion, physiotherapy, and rehabilitation settings, presently there is a lack of data on the effect of these manoeuvres on trunk muscles activity during dynamic exercises.

The purpose of this study was to evaluate peculiarities of trunk muscles electrical activity in young adults in the course of static and dynamic trunk muscles strengthening exercises using different lumbar spine stabilization manoeuvres. Drawing upon previous studies, we hypothesized that relative to exercises with AB manoeuvre, during exercises with AH manoeuvre, there would be higher electrical activity in deep abdominal muscles and lower electrical activity in surface abdominal muscles.

## Methods

### Subjects

Inclusion criteria for this study consisted of females and males, between 18 and 44 years of age with no experience of acute pain on the testing day, no history of trauma and pain localized around knees, hips, elbows or shoulders over one-year period. All the experimental procedures were approved by the local research ethics canter of Lithuanian Health Sciences University (BEC SR(M)-177) and were carried out according to the Helsinki Declaration.

We invited volunteers to participate in this study in two months’ period. Consistent with the sample size in previously similar work [2, 21, 31, 32, 33], a total of 20 (ten males and ten females) young adult subjects (M_age_ = 25.5 SD = 2.91) participated in this study. 11 subjects (5 males and 6 females) reported no history of pain, 9 subjects (5 males and 4 females) reported LBP within the last three months. Pain intensity ranged between 2–5 points (mild to moderate pain) on the Numeric Rating Scale of Pain (NRS Pain). All the subjects signed an informed consent form prior to taking part in the study. Subjects provided information about LBP location, duration, and type. We used Oswestry Disability Index (ODI) and NRS Pain for pain ratings. Physical Activity Readiness Questionnaire (PAR-Q) was administered to ensure for similar levels of physical activity among subjects (see Table 1).

**Table 1:** Pain characteristics and Oswestry Disability Index scores. Note: values are presented as Median value: Xme (minimum value (Xmin)—maximum value (Xmax)). LBP—low back pain; NRS- Numeric Rating Scale for Pain; ODI—Oswestry Disability Index.

Participants	Experienced LBP in the last three months	Median value of NRS	Median value of ODI
**Females (n = 10)**	n = 5 (45.45%)	2(1–4)	10(0–14)
**Males (n = 10)**	n = 6 (54.54%)	2.5(2–5)	7(2–18)
**All in (n = 20)**	**n = 11 (55%)**	**2(1–5)**	**10(0–18)**

*Note*: NRS–Numeric Rating Scale for Pain; ODI–Oswestry Disability Index.

### Evaluation of trunk muscles electrical activity

Noraxon Telemyo twelve channel electromyography device (Noraxon USA, Inc.) was used to record EMG data. FIAB (Spain; FOAM; rectangular shape 21x41 mm diameter; 22 mm inter-electrode distance; Ag/AgCl; F3010 type) disposable, bipolar electrodes with solid gel helped record EMG data. The electrodes were prepared by scrubbing the skin with fine sand paper, drying the skin with isopropyl alcohol, and shaving the hair. Surface electrode pairs were applied on the prepared skin. Electrodes were applied on RA, EO, IO and ES muscles on the right side only because previous studies have shown a symmetry of trunk muscles EMG activity [34]. Within the present protocol, electrodes were placed based on previously validated protocols [13, 24, 35]. Specifically, they were positioned to match the direction of the muscle fibers. RA electrodes were aligned vertically and at the center of the muscle belly, 2 cm lateral and 3 cm superior to the umbilicus. EO electrodes were positioned obliquely approximately 45° near the level of umbilicus, midway between rib cage and anterior superior iliac spine (ASIS). IO electrodes were located horizontally 2 cm inferomedial to the ASIS, within a triangle confined by the inguinal ligament, lateral border of the rectus sheath, and a line connecting the ASIS. Finally, ES electrodes were set 3cm lateral to the level of L4/L5 spinous process.

### Evaluation of maximal voluntary isometric contraction

**Fig 1:**
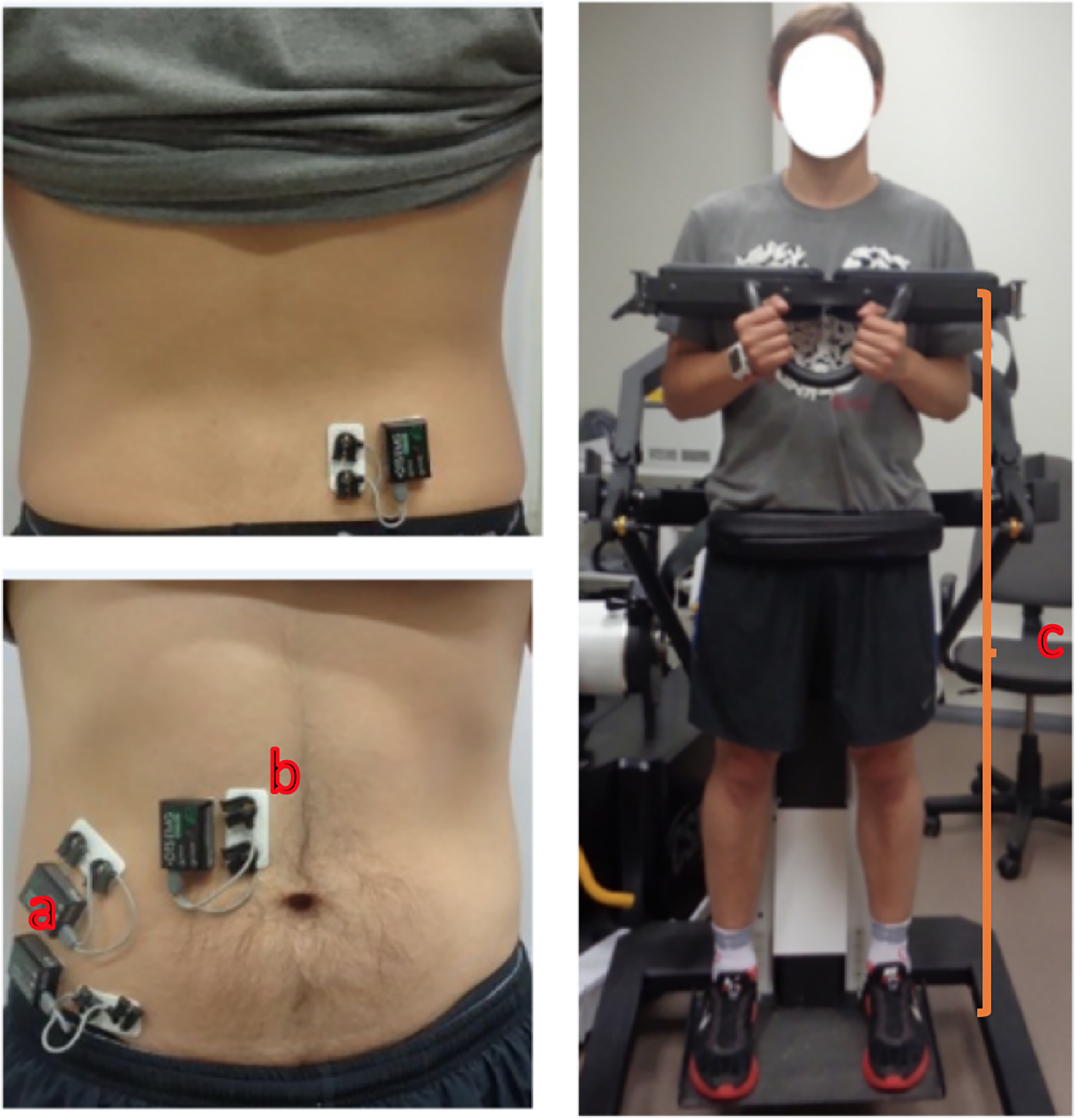
Preparation and the procedure of maximal isometric voluntary contraction with electromechanical dynamometer and surface electrodes. a) Direct transmission system (DTS) sensors, b) EMG electrodes c) electromechanical dynamometer.

For the normalization of EMG signals, MVC for each muscle was measured and EMG signal amplitudes were recorded. An electromechanical dynamometer (Humac Norm; CSMI, Stoughton, MA) was used. The measurements were made in standing position, with two belts fixed around pelvis and under the shoulders (Fig 1). Initially, the calibration of the device was performed and subjects were required to perform trunk flexion, trunk extension and trunk side-flexion, while the maximum amplitudes of these movements were measured. Calibration was performed before each measurement of maximum force.

During the MVC measurements, subjects were instructed to perform a maximum isometric trunk flexion, trunk extension and side flexion to the left side and to the right side. In order to help maximize the output, all subjects were provided with verbal encouragement in the course of their performance. Three repetitions of each movement were performed, each of which lasted about 5 seconds. There were 30 seconds pause between repetitions. Both, the amplitudes (V) of EMG signals of the RA, EO, IO and ES muscles and maximum force (N) were recorded. The average electromyographic amplitudes obtained at maximum force were used to normalize electromyographic data during exercises. Electromyographic data was expressed as a percentage (%) of electromyographic amplitudes corresponding to maximum muscular force.

### Performance of static and dynamic exercises

**Fig 2:**
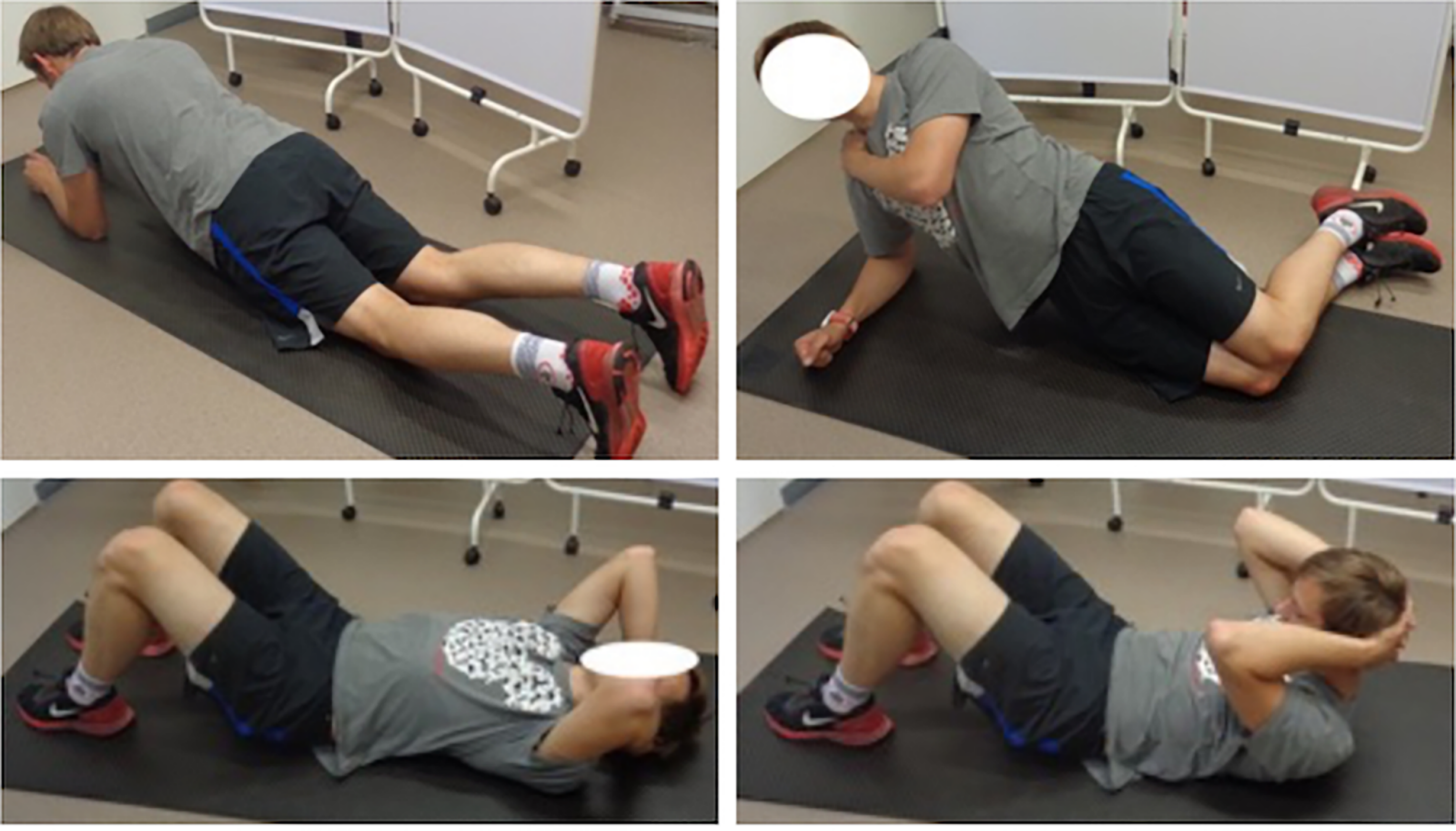
Performed exercises in following the same sequence. From the top Kneeling plank exercise, Side-bridge exercise and Curl-up (stabilization manoeuvre and upper torso was curled up).

Subjects were instructed to perform exercises which are frequently used in rehabilitation and athletic programs. These included planks [14, 29], side-bridges [31], and curl-ups [32]. They were performed with two manoeuvres—AH and AB. Each subject took about 15 minutes to learn how to perform exercises and manoeuvres correctly. None of the subjects had previously practiced the AH or AB manoeuvres. Each exercise was repeated 3 times. Rest interval between repetitions was at least 30 seconds. Rest interval between exercises was at least 2 minutes. A metronome was used to help subjects complete their exercise in time. All the exercises were performed following the same sequence (see Fig 2):

1. Kneeling plank exercise: in a kneeling plank position subjects were instructed to perform stabilization manoeuvre within 2 seconds and keep initial position for 5 more seconds without holding their breath.
2. Side-bridge exercise: initially, side-bridge was performed on the right side then on the left side [31]. In a kneeling side-bridge position stabilization manoeuvre was performed within 2 seconds and the position was kept for 5 more seconds without holding the breath.
3. Curl-up: initially, subjects had to maintain starting position and within 2 seconds perform stabilization manoeuvre, then in 2 seconds the upper torso was curled up and kept for 1 second. Next, they had to come back to the starting position within 2 seconds. During this exercise, ES muscle activity was not measured because the pressure on the back of electrodes and the sensor would have caused discomfort to subjects which would impact the measurement of the EMG signal.

### Data analysis

EMG signal normalization was computed using Myomuscle software (Noraxon MR 3.6). EMG data were band-pass filtered (the frequency range 5–500 Hz), then rectified and smoothed. The amplitude of EMG data was normalized using the mean dynamic activity method [36]. For further analysis of processed EMG data, special markers in the software were used to help delimit individual phases of exercise and for computing the results in different phases of exercise. We analysed static exercises’ data between seconds 3–7. Dynamic exercises were analysed in separate phases: isometric muscles activation, lifting and landing. For each exercise, the EMG data was normalized for each muscle and expressed as a percentage of muscle activity at the MVC. For statistical analysis, the mean of normalized EMG data was derived from three trials for each exercise.

Statistical analysis was performed using SPSS 23.00 statistical analysis package. The results are presented as a median Xme (minimum value (Xmin)—maximum value (Xmax)). A non-parametric Wilcoxon test was used to compare the two dependent samples. With regards to the relationship between quantitative variables, Spearman’s correlation coefficient (r) was computed. A correlation coefficient of |r|<0.3 was considered weak, while 0.3≤|r|≤0.7 was considered moderate, and |r|>0.7 was considered strong. We set the significance level at *p* < 0.05.

### Results

**Fig 3:**
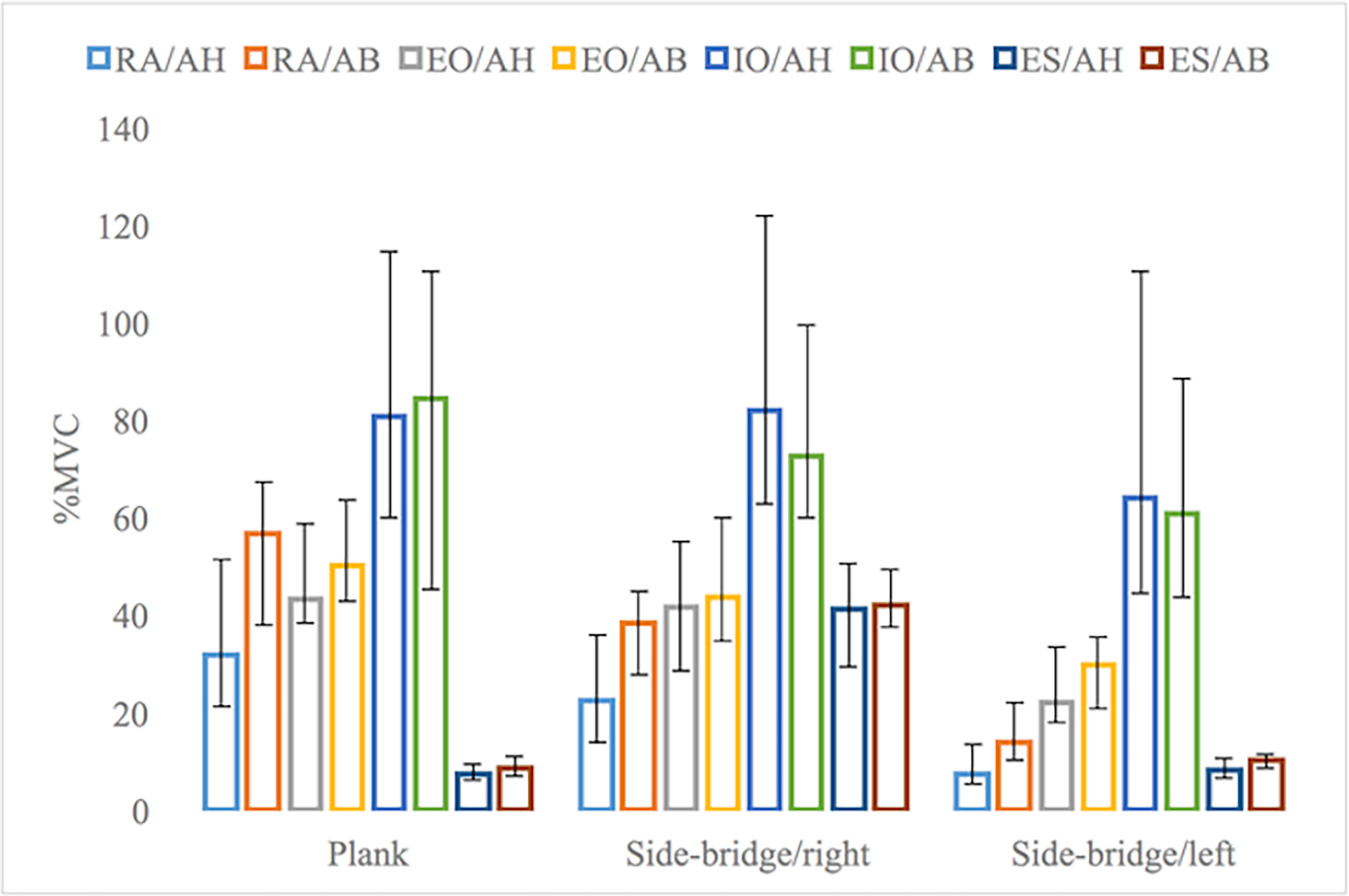
Trunk muscles’ activity during static exercises. Notes: RA–rectus abdominal muscle, EO–external obliques muscles, IO–internal obliques muscles, ES–erectus spine muscles, AB–abdominal bracing, AH–abdominal hollowing. The results are presented as a median and non parametric confidence interval of median.

During kneeling plank exercise, RA muscle electrical activity performing AB manoeuvre was significantly higher (Z = -3.72 p = 0.001). No significant differences were found in electrical activity of EO, IO and ES muscles while using different stabilization manoeuvres during kneeling plank exercise (Z = -1.489; p = 0.136), (Z = -1.891; p = 0.06), Z = -1.551; p = 0.121), respectively, (see Fig 3).

Side-bridge exercise performed on the right side showed significantly higher RA muscle electrical activity with AB manoeuvre compared to AH manoeuvre (Z = -2.17; p = 0.03). During this exercise, median value of EO muscle electrical activity was higher with AB than with AH manoeuvre, but this difference was not significant (Z = -1.027; p = 0.305). No significant difference was observed in IO muscle nor ES muscle performing side-bridge exercise with AB and with AH manoeuvres (Z = -1.904; p = 0.057), (Z = -1.699; p = 0.89), respectively.

Similar the right side, during side-bridge exercise performed on the left side, RA muscle electrical activity with AB manoeuvre was significantly higher than with AH manoeuvre (Z = -3.40; p = 0.001). Unlike the right side, significant differences were also found in electrical activity of EO and ES muscles in side-bridge exercise on the left side with different stabilization manoeuvres (Z = -2.535; p = 0.011; Z = -2.178; p = 0.029). The electrical activity of these muscles was significantly higher with AB manoeuvre than AH manoeuvre. Although the median value for IO muscle electrical activity was higher with AH than with AB manoeuvre, this difference was not significant (Z = -0.784; p = 0.433).

**Fig 4:**
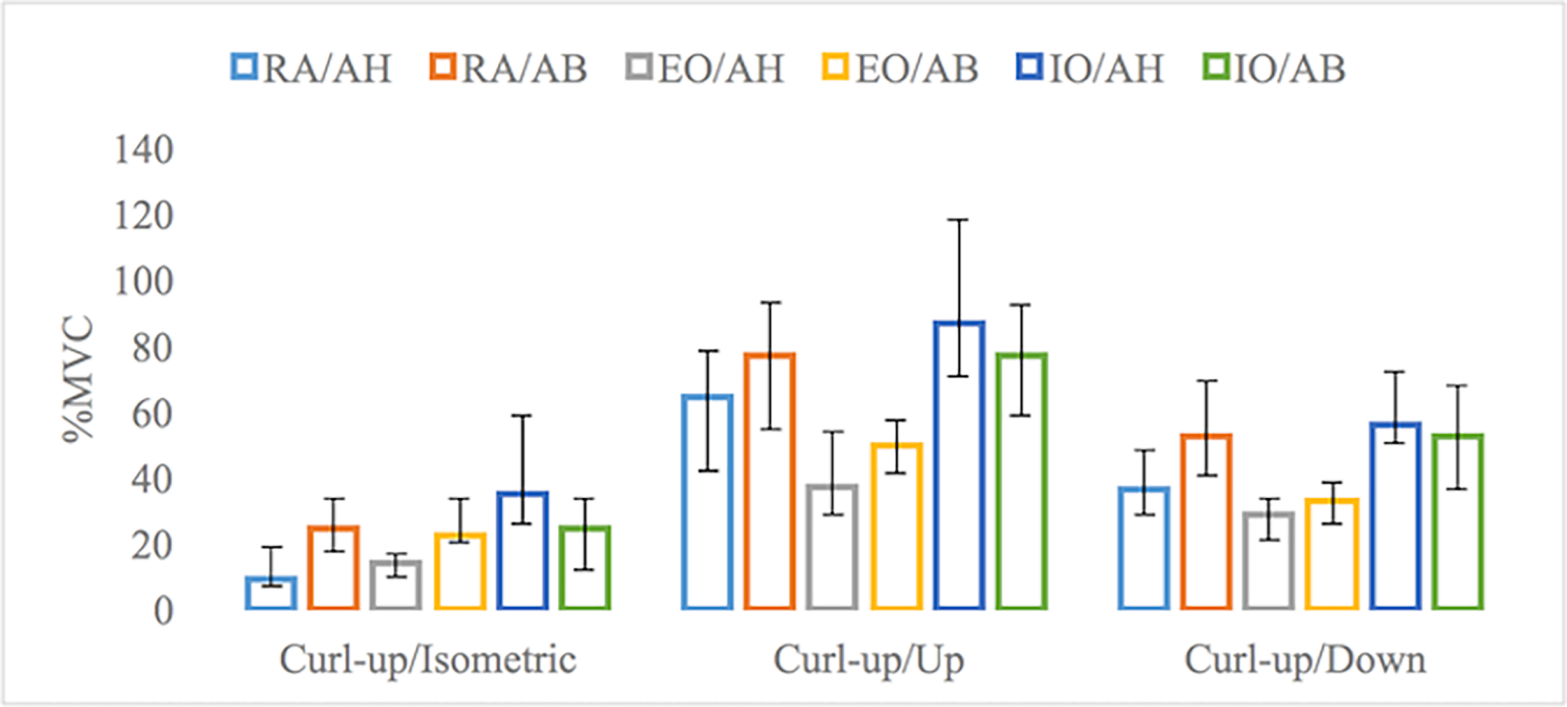
Trunk muscles’ activity during dynamic exercises. Notes: RA–rectus abdominal muscle, EO–external obliques muscles, IO–internal obliques muscles, ES–erectus spine muscles, AB–abdominal bracing, AH–abdominal hollowing. The results are presented as a median and non parametric confidence interval of median.

In the isometric muscles activation phase of curl-up exercise, median value of RA muscle electrical activity was significantly higher with AB manoeuvre compared to AH manoeuvre (Z = -2.591; p = 0.010). No significant differences were found between median values of EO and IO muscles electrical activity with different stabilization manoeuvres (Z = -1.941; p = 0.052; Z = -1.008; p = 0.313). In the lifting phase of curl-up exercise, median value of RA muscle activity with AB was significantly higher than with AH (Z = -2.315; p = 0.021). Median value of IO muscle activity with AH was significantly higher than with AB (Z = -3.230; p = 0.001). However, median value of EO muscle electrical activity did not differ significantly between manoeuvres (Z = -1.307; p = 0.191). In the landing phase of curl-up exercise, median value of RA muscle electrical activity with AH was significantly lower than with AB manoeuvre (Z = -2.651; p = 0.008). IO muscle electrical activity was significantly higher with AH than with AB manoeuvre (Z = -2.725; p = 0.006). Finally, no significant difference was found between these manoeuvres in median value of EO muscle electrical activity during the landing phase of the curl-up exercise (Z = -1.176; p = 0.240), see Fig 4 for more details.

Comparison of muscles’ electrical activity in static and dynamic exercises indicated highest activity values for RA in the lifting phase of the curl-up exercise with AB manoeuvre. RA electrical activity was significantly higher compared to plank exercise performed with AB manoeuvre (Z = -2.949; p = 0.003). The lowest activity of RA muscle was during side-bridge exercise on the left side with AH manoeuvre. RA muscle activity was significantly lower in side-bridge than in curl-up exercise in the isometric muscle activation phase with AH manoeuvre (Z = -2.222; p = 0.026). The highest activity of EO and IO muscles between static and dynamic exercises showed no differences (Z = -1.381; p = 0.167 and Z = -0.448; p = 0.654). EO and IO muscles’ activation were significantly lower during dynamic exercise than during static exercise (Z = -2.054; p = 0.040 and Z = -2.128; p = 0.033). Data in Table 2 indicates differences in specific muscle activations during exercises (showing highest and lowest activity).

**Table 2:** Comparison of trunk muscles’ electrical activity in static and dynamic exercises—highest and lowest activity values. Notes: RA–rectus abdominal muscle, EO–external obliques muscles, IO–internal obliques muscles, ES–erectus spine muscles, AB–abdominal bracing, AH–abdominal hollowing.

	Static exercises		Dynamic exercises
Maximal activity of RA	**Plank exercise + AB manoeuver**	***	**Curl-up exercise lifting phase + AB**
Minimal activity of RA	Side-bridge on the left side + AH manoeuver	***	Curl-up exercise isometric phase + AH manoeuver
Maximal activity of EO	**Plank exercise + AB manoeuver**		Curl-up exercise lifting phase + AB manoeuver
Minimal activity of EO	Side-bridge on the left side + AH manoeuver	***	Curl-up exercise isometric phase + AH manoeuver
Maximal activity of IO	**Side-bridge on the right side + AH manoeuver**		**Curl-up exercise lifting phase + AH manoeuver**
Minimal activity of IO	Side-bridge on the left side + AB manoeuver	***	Curl-up exercise isometric phase + AH manoeuver
Maximal activity of ES	Side-bridge on the left side + AB manoeuver		
Minimal activity of ES	Plank exercise + AH manoeuver		

Notes

***—p<0.05

Highlighted results indicate muscle electrical activity higher than 50% of MVC in the course of a particular exercise.

There were no significant correlations between pain intensity according to NRS Pain and trunk muscles electrical activity (%). Additionally, no significant correlations between trunk muscles’ electrical activity and subjects with and without LBP were found. Consequently, no further analysis was performed. A moderately significant direct correlation was found between the percentage of ODI and EO muscle electrical activity in all phases of curl-up exercise with AH manoeuvre. And statistically significant direct dependence relationship was found between the percentage of ODI and EO muscle activity during side-bridge exercise on the right side with AB manoeuvre. Table 3 depicts statistically significant interfaces.

**Table 3:** Correlation between the percentage of ODI and %MVC of EO muscle. Notes: ODI—Oswestry Disability Index; MVC—maximal isometric voluntary contraction; EO—external oblique muscles.

	%MVC activity of EO performing Curl-up exercise isometric phase with AH manoeuver	%MVC activity of EO performing Curl-up exercise lifting phase with AH manoeuver	%MVC activity of EO performing Curl-up exercise landing phase with AH manoeuver	%MVC activity of EO performing Side-bridge exercise on the right side with AB manoeuver
ODI %	r = 0,582 p = 0,007	r = 0,526 p = 0,017	r = 0,590 p = 0,006	r = 0,481 p = 0,032

### Discussion

Our findings indicate that although surface muscles are more activated with AB manoeuvre exercises, deep abdominal muscles get more activated in exercises with AH manoeuvre. Consistent with the present findings, others have also shown highest activity of RA muscles during curl-up exercises [35]. Present findings indicated that IO activity was higher than 50% of MVC, in contrast to some of previous findings which indicated it as lower than 50% of MVC. These discrepancies can be due to the differences in research methodology. Specifically, no stabilization manoeuvres were used in these studies [35] or mean of muscles activity were not analysed by combining all phases into one, without separating them as in the present research [32].

From a methodological standpoint, in order to facilitate task-performance in our study, we have modified plank exercises. As such, our kneeling planks were weight bearing through the knees rather than the toes.

Present findings also indicate that electrical IO muscle activity during kneeling plank position with AH was highest, while surface muscle activity was minimal. These results are further consistent with previous work that did not use facilitated plank protocols [20]. However, some have noted that EO muscles are most active during plank exercise. This difference was observed when plank exercise was performed without using any stabilizing manoeuvre [33, 37]. To that end, Marshall et al. [13] showed no statistically significant differences in the evaluation of trunk muscle activity during trunk muscles strengthening exercises between patients with and without LBP. Nevertheless, Kang et al. [38] showed a decrease in trunk muscle activity in patients with LBP during plank exercises compared to healthy subjects.

There is also some evidence that surface abdominal muscle (EO, RA) activity is the highest during dynamic abdominal muscle strengthening exercises (sit-up, curl-up) and the ES muscle is the highest during side-bridge exercise [21]. In the present study, AH and AB manoeuvres were performed as separate exercises and electrical activity of IO muscle was the highest during these exercises. In our study, RA activity was also higher in dynamic curl-up exercise than in static exercises, but there was no significant difference between EO and IO muscles activity in static and dynamic exercises.

There are several limitations to our study. First, we would like to note that more precise data from deep abdominal muscles could be obtained via placement of intramuscular electrodes. This method however was not possible in our study given the dynamical nature of our protocol. Second, we used surface EMG electrodes for deep (IO) abdominal muscle activity. Although electrodes were arranged in accordance with IO muscle fibers, EMG signals might have been obtained from both muscles: IO and TrA. Also, we have not performed any biomechanical measurements for compression or shear forces that affect the lumbar spine during exercises. This data would be helpful in assessing the tissue load and safety aspects of the various movements and using different manoeuvres. Finally, our study did not evaluate the activity of transversus abdominus, lumbar multifidus muscles, and pelvic floor muscles which are also responsible for trunk stability [2].

Also, lastly, the results from this study may not apply to all age groups. These findings may not facilitate translation between participants with different ages and fitness levels. Further studies must be conducted in order to find out if such conclusions are appropriate for the participants of different age groups and fitness levels. To that end, further cross-sectional studies with different age groups may help strengthen our conclusions. Similarly, longitudinal studies examining specific age ranges too could help confirm and solidify our findings.

To conclude, the present study examined the effects of AH and AB manoeuvres during static and dynamic exercises. On the basis of our findings, the AH manoeuvre enhanced electrical activity of deep abdominal muscles with minimal surface muscles activation, while AB manoeuvre promoted a broader muscle activation during static and dynamic exercises. Although different studies made use of these manoeuvres in different positions [20–24, 32] we found it of ample importance not only to know how the muscle activation evolves with these stabilisation manoeuvres in different positions, but also to unravel the activation during static and dynamic exercises. Consequently, the unique contribution of this work lies in the use of stabilization manoeuvres not only in different positions but also during static and dynamic exercises. Moreover, to the best of our knowledge, this attempt is amongst the first to evaluate muscles activity in a modified kneeling plank position (with weight bearing through the knees rather than the toes). Therefore, present results pertaining to AH and AB manoeuvres are important both for the advancement of the field and also for improving clinical practice including that of clinicians, physical therapy practitioners, athletic trainers and other specialists involved with exercise. It appears that based on the evidence presented herein, practitioners can design interventions to integrate AH manoeuvre for benefiting persons with lack of lumbar motor control. Additionally, these results may benefit practitioners to alter motion patterns and AB manoeuvre to improve spinal stability in athletes and healthy individuals. Effectiveness of these strategies for decreasing events of lumbar injury and pain should be therefore further explored and evaluated.
